# A recurrent *ABCC2* p.G693R mutation resulting in loss of function of MRP2 and hyperbilirubinemia in Dubin-Johnson syndrome in China

**DOI:** 10.1186/s13023-020-1346-4

**Published:** 2020-03-18

**Authors:** Lina Wu, Yanmeng Li, Yi Song, Donghu Zhou, Siyu Jia, Anjian Xu, Wei Zhang, Hong You, Jidong Jia, Jian Huang, Xiaojuan Ou

**Affiliations:** Liver Research Center, Experimental Center, Beijing Friendship Hospital, Capital Medical University, 95 Yong-An Road, Beijing, 100050 China

**Keywords:** Dubin-Johnson syndrome, Adenosine triphosphate-binding cassette subfamily C member 2, Multidrug resistance-associated protein 2, Missense mutation, Biological function

## Abstract

**Background:**

Dubin-Johnson syndrome (DJS) is a rare autosomal recessive disorder characterized by predominantly conjugated hyperbilirubinemia that is caused by pathogenic mutations in the adenosine triphosphate-binding cassette subfamily C member 2 (*ABCC2*) gene, which encodes multidrug resistance-associated protein 2 (MRP2). However, little is known about the causative mutation of DJS in China. Recently, we have reported *ABCC2* p.G693R mutation in two unrelated cases. In the present study, we investigated the pathogenicity of the *ABCC2* p.G693R mutation in DJS in China.

**Methods:**

Clinical and genetic analysis was conducted for the two patients with the *ABCC2* p.G693R mutation. Whole exome sequencing for mutations in other known hyperbilirubinemia-related genes was conducted for the cases with *ABCC2* p.G693R. Expression and cellular localization of the mutant MRP2 p.G693R were analyzed by Western blotting and immunofluorescence assay, respectively. Organic anion transport activity was evaluated by the analysis of glutathione-conjugated-monochlorobimane.

**Results:**

The two DJS patients with *ABCC2* p.G693R mutation, which was conserved among different species, showed typical hyperbilirubinemia phenotype. No pathogenic mutation was identified in the other known hyperbilirubinemia related genes. Functional studies in three cell lines showed that the expression, localization and the organic anion transport activity were significantly compromised by MRP2 p.G693R mutation compared with wild-type MRP2.

**Conclusions:**

The recurrent *ABCC2* p.G693R mutation is associated with loss of function of the MRP2 protein and may result in hyperbilirubinemia in DJS in China.

## Background

Dubin-Johnson syndrome (DJS) is an autosomal recessive disorder which was first described in 1954 [1]. As a rare disorder affecting both genders, DJS has been identified in all nationalities and races. Its incidence in Sephardic Jews is approximately 1 in 3000 [2]. The syndrome is characterized by predominantly conjugated hyperbilirubinemia, which is caused by impairment in the transfer of non-bile acid organic anions from hepatocytes into canaliculi [3].

The adenosine triphosphate-binding cassette subfamily C member 2 (*ABCC2*) gene, located on chromosome 10q24, encodes the multidrug resistance-associated protein 2 (MRP2). Comprised of 1545 amino acids, this protein belongs to an integral membrane glycoprotein family [4]. MRP2 traffic from the endoplasmic reticulum to the canalicular membrane of hepatocytes where it functions and then relocalizes back to endosomal vesicles for recycling [5]. This protein is a non-bile acid organic anion transporter and mediates the active transport of conjugate compounds with glutathione or glucuronate from the cytoplasm of hepatocytes into the canaliculi [5].

According to Human Gene Mutation Database (HGMD; www.hgmd.cf.ac.uk), a total of 68 variants in the *ABCC2* gene including missense, nonsense, deletions and splice site mutations have been identified in DJS patients. However, no hotspot mutations have been identified in the *ABCC2* gene. The majority of the DJS-causing mutations in *ABCC2* are related to defects in MRP2 protein synthesis, localization or secretion activities. Some mutations may cause rapid degradation of the mRNA, mislocalization of protein or decreased organic anion transport activity [6]. *ABCC2* mutations have been identified in DJS patients worldwide. However, less is known about the causative mutation of DJS in China.

Recently, we identified *ABCC2* p.G693R mutation in our previous study [7]. Therefore, in the present study, we investigated the frequency of *ABCC2* p.G693R in Chinese DJS patients and examined the pattern and biological consequences of the *ABCC2* p.G693R mutation, focusing on their effects on protein maturation, localization and transport activity.

## Methods

### Study population

From the China Registry of Genetic/Metabolic Liver Diseases (ClinicalTrials.gov identifier, NCT03131427), a total of 14 patients suspected with DJS, who had biochemical evidence of fluctuating predominantly conjugated hyperbilirubinemia with or without family history, were initially included in the present study between June 2015 and December 2017. Whole blood samples from the 14 patients were collected and stored at − 20 °C for Sanger sequencing. 7 out of the 14 patients had *ABCC2* gene mutations, and 2 of the 7 patients had *ABCC2* p.G693R mutation [7].

The study was conducted in accordance with the Declaration of Helsinki. The Clinical Research Ethics Committee of Beijing Friendship Hospital, Capital Medical University approved the study protocol (No. 2019-P2–217-02). All patients provided written informed consent.

### Clinical and genetic analysis of the two patients with the *ABCC2* p.G693R

The clinical manifestation of the patients with the *ABCC2* p.G693R, including age of onset, duration of jaundice, aggravating or relieving factors was recorded. Past medical history including drug or toxin exposure, alcohol intake, and family history of jaundice or other liver diseases were collected.

Relevant laboratory data of the two patients with the *ABCC2* p.G693R were analyzed, including complete blood count, liver function tests, renal function tests and electrolytes, coagulation profile. Abdominal ultrasonography was done to exclude obstruction or dilation of the hepatobiliary tract exist and transient elastography (FibroScan) was conducted to evaluate the liver stiffness.

Conservative analysis was performed by http://genome.ucsc.edu/. Aligned amino acid sequences of human, rhesus, mouse, dog, elephant, chicken, xenopus tropicalis, zebrafish, and lamprey MRP2 with mutation p.G693R loci were analyzed.

### Analysis of mutation in other known hyperbilirubinemia genes by whole exome sequencing

Approximately 1 μg of genomic DNA was used to construct a whole exome library with an insert size of 150–200 bp by an exome capture strategy using a GenCap custom exome enrichment kit (MyGenostics, Beijing, China). Paired-end 100 bp raw reads from each enriched library were generated with an Illumina HiSeq 2000 platform (Illumina, San Diego, USA) according to the manufacturer’s protocol. The paired-end reads were aligned against NCBI build 37 of the human genome using Burrows Wheeler Aligner. With the GenomeAnalysis Toolkit (GATK4.1.2.0, https://software.broadinstitute.org/gatk/download/), duplicate reads were marked; local indel realignment was performed, and base quality scores were recalibrated for each sample. The identified potential pathogenic variants were confirmed by Sanger sequencing.

Polyphen-2 (http://genetics.bwh.harvard.edu/pph2), SIFT (https://sift.bii.a-star.edu.sg/) and MutationTaster (http://www.mutationtaster.org/) were used to predict the biofunctional consequence of the identified variants.

### Functional analysis of *ABCC2* p.G693R mutant

#### Construction of the *ABCC2* p.G693R mutant

To create the *ABCC2* wild-type plasmid, we amplified *ABCC2* from human cDNA and cloned it into *Hind* III*/Not* I sites of the pcDNA3.1 vector along with the N-terminal flag tag [8]. The *ABCC2* p.G693R constructs were generated using the Gene Tailor Site-Directed Mutagenesis System (Invitrogen, Waltham, MA, USA).

#### Cell culture and transfection

Human embryonic kidney (HEK) 293A cells and human liver cancer cell lines Huh-7 and HepG2 were obtained from the Cell Resource Center of the Chinese Academy of Medical Science (Beijing, China). The Cell lines were cultured in Dulbecco’s modified Eagle’s medium supplemented with 10% fetal bovine serum, 100 units/ml penicillin and 100 units/ml streptomycin. Then the cells were transfected with plasmids expressing *ABCC2* wild-type or *ABCC2* p.G693R by Lipofectamine 3000 (Invitrogen, Carlsbad, USA) according to the manufacturer’s instructions; the culture medium was changed at 6 h after transfection. Finally, the cells were harvested 24 or 48 h after transfection.

#### Measurement of MRP2 expression by Western blotting

The cells were lysed in RIPA buffer with proteinase inhibitor. After centrifugation, the supernatant was used for western blot analysis. Proteins were separated by SDS-PAGE (8%) and transferred to nitrocellulose membranes; the membranes were incubated with anti-MRP2 monoclonal antibodies M2III-6 (1:200; sc-59,608; Santa Cruz Biotechnology, Dallas, TX; a mouse monoclonal antibody raised against a C-terminal region of MRP2 of human origin) or anti-GAPDH antibodies (1:5000; Santa Cruz Biotechnology) overnight at 4 °C, followed by incubation with horseradish peroxidase (HRP)-conjugated goat anti-mouse antibody (1:5000 dilution; Santa Cruz Biotechnology) for 1 h at 37 °C. Immunocomplexes on the membrane were visualized using Immobilon Western Chemiluminescent HRP Substrate (Millipore, Billerica, USA) and Image Lab Software (BIO-RAD, Hercules, USA).

#### Measurement of subcellular localization by indirect immunofluorescence staining

Immunofluorescence analysis was performed as described previously [9]. The cells were incubated with a primary antibody directed against rabbit anti-MRP2 (ab172630; Abcam) and mouse anti-flag (#8146; Cell Signaling technology) at 4 °C overnight. After three washes with phosphate-buffered saline for 5 min each, the cells were incubated with anti-rabbit Alex 647 and anti-mouse Alex 488-conjugated secondary antibodies (1:200; Invitrogen) for 1 h at room temperature. For F-actin staining, cells were incubated with FITC-conjugated phalloidin at 50 μg/ml (P5282; Sigma) for 40 min. Then cells were mounted on a slide in mounting medium (Molecular Probes). Finally, the cells were visualized and photographed using an FV 300 confocal microscope (Olympus, Tokyo, Japan).

#### Measurement of organic anion transport activity by export of glutathione conjugated monochlorobimane (GS-MCLB) assay

MCLB is an organic anion transport substrate for MRP2 and has absorption/emission maximal ~ 394/490 nm. MCLB (M1381MP, Thermo, USA) transport study was conducted as described by Terlouw et al [10]. The cells were pre-incubated with 0.2 mmol/L MCLB in medium for 30 min on ice. The medium was replaced with fresh Hank’s medium and incubated at 37 °C. At different time points, medium was collected and the GS-MCLB in the medium was measured by the fluorescence method with a spectrophotometer.

### Statistical analysis

All experiments were carried out at least three times. Statistical analyses were performed using SPSS V12.0 software. Results were expressed as mean ± standard deviation (SD). Continuous variables were analyzed using the Student’s test. A two-sided *P* value of < 0.05 was considered statistically significant.

## Results

### Clinical and genetic profiles of the DJS patients with *ABCC2* p.G693R mutation

**Fig. 1:**
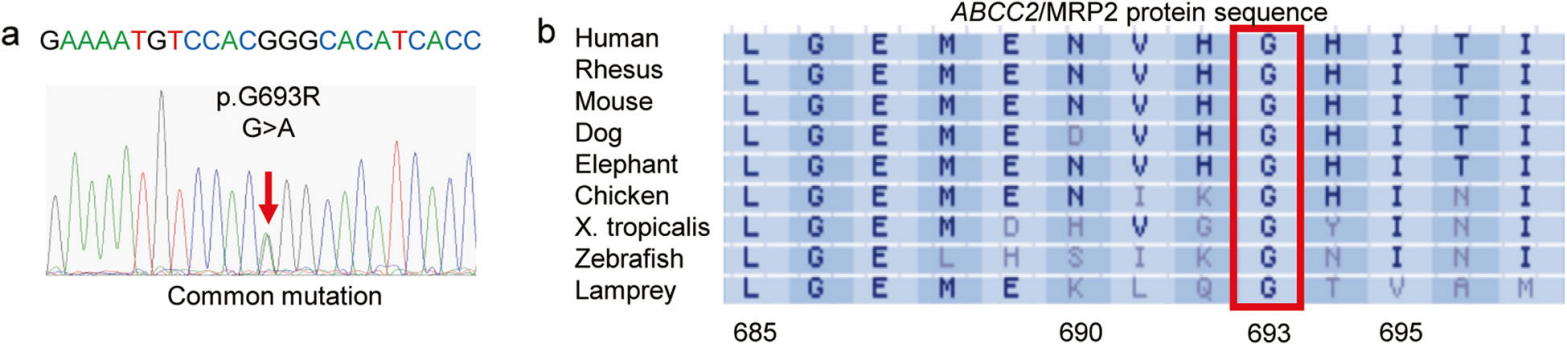
Mutation analysis of the two DJS cases with *ABCC2* p.G693R. **a** Sequencing of the heterozygous missense mutation c.2190G > A, p.G693R in exon 16 in both cases. **b** Aligned amino acid sequences of human, rhesus, mouse, dog, elephant, chicken, xenopus tropicalis, zebrafish, and lamprey MRP2 with mutation p.G693R flanking regions. The position of the p.G693R mutation is indicated by the red box. Alignment was performed by http://genome.ucsc.edu/

For the two unrelated patients with DJS, patient No. 1 (female, 21 years old) and patient No. 2 (male, 39 years old) harbored the heterozygous allelic variant c.2190G > A in exon 16 of *ABCC2*, which resulted in the substitution of arginine for glycine at position 693 (p.G693R) (Fig. 1a). Sequence comparison showed that amino acid 693 of MRP2 was conserved among different species (Fig. 1b). The two patients harbored the variant of p.G808V or p.R529Q in another allele respectively.

The clinical features of these two patients are shown in Table 1. Total bilirubin and direct bilirubin were 94 μmol/L and 54 μmol/L in patient No.1, and 97 μmol/L and 49 μmol/L in patient No.2 respectively. The aminotransferase, alkaline phosphatase, γ-glutamyl transpeptidase, total bile acid and albumin in liver function tests were within the normal range. Abdominal ultrasonography showed no obstruction or dilation of the hepatobiliary tract in both patients. Liver stiffness by FibroScan was 3.5 Kpa in patient No.1, but not available in patient No. 2.

**Table 1:** Clinical characteristics and non-synonymous variants identified in DJS patients with *ABCC2* p.G693R mutation

Patient Number	Age (year)	Sex	Variants	TB (μmol/L)	DB (μmol/L)	ALT (U/L)	AST (U/L)	ALP (U/L)	GGT (U/L)	TBA (μmol/L)	ALB (g/L)	PTA (%)	Abdominal ultrasonography	Liver stiffness (Kpa)
1	21	Female	p.G693R (c.2190G > A), p.R529Q (c.1586G > A)	94	54	48	28	79	32	2.9	45	98	Normal	3.5
2	39	Male	p.G693R (c.2190G > A), p.G808V (c.2536G > T)	97	49	43	17	78	23	2.8	46	109	Hepatic hemangioma,cholecystolithiasis	NA

*DJS* Dubin-Johnson syndrome, *TB* total bilirubin, *DB* direct bilirubin, *ALT* alanine aminotransferase, *AST* aspartate aminotransferase, *ALP* alkaline phosphatase, *GGT* γ-glutamyl transpeptidase, *TBA* total bile acid, *ALB* albumin, *PTA* prothrombin activity, *NA* not available

Results from whole exome sequencing revealed additional p.R110Q (c.329G > A) mutation in the gene 3β-hydroxysteroid dehydrogenase type 7 (*HSD3B7*), which is related with bile acid synthesis type I, in patient No. 2 with *ABCC2* p.G693R mutation, with allele frequency of *HSD3B7* p.R110Q is 5.80E-05 in East Asian and 3.00E-04 in total, respectively.. However, software prediction demonstrated that the missense mutation *HSD3B7* p.R110Q is benign or tolerable. In patient No. 1 with *ABCC2* p.G693R mutation, whole exome sequencing revealed no mutations in the other known hyperbilirubinemia-related genes.

### MRP2 expression was decreased in cell lines expressing MRP2 p.G693R

**Fig. 2:**
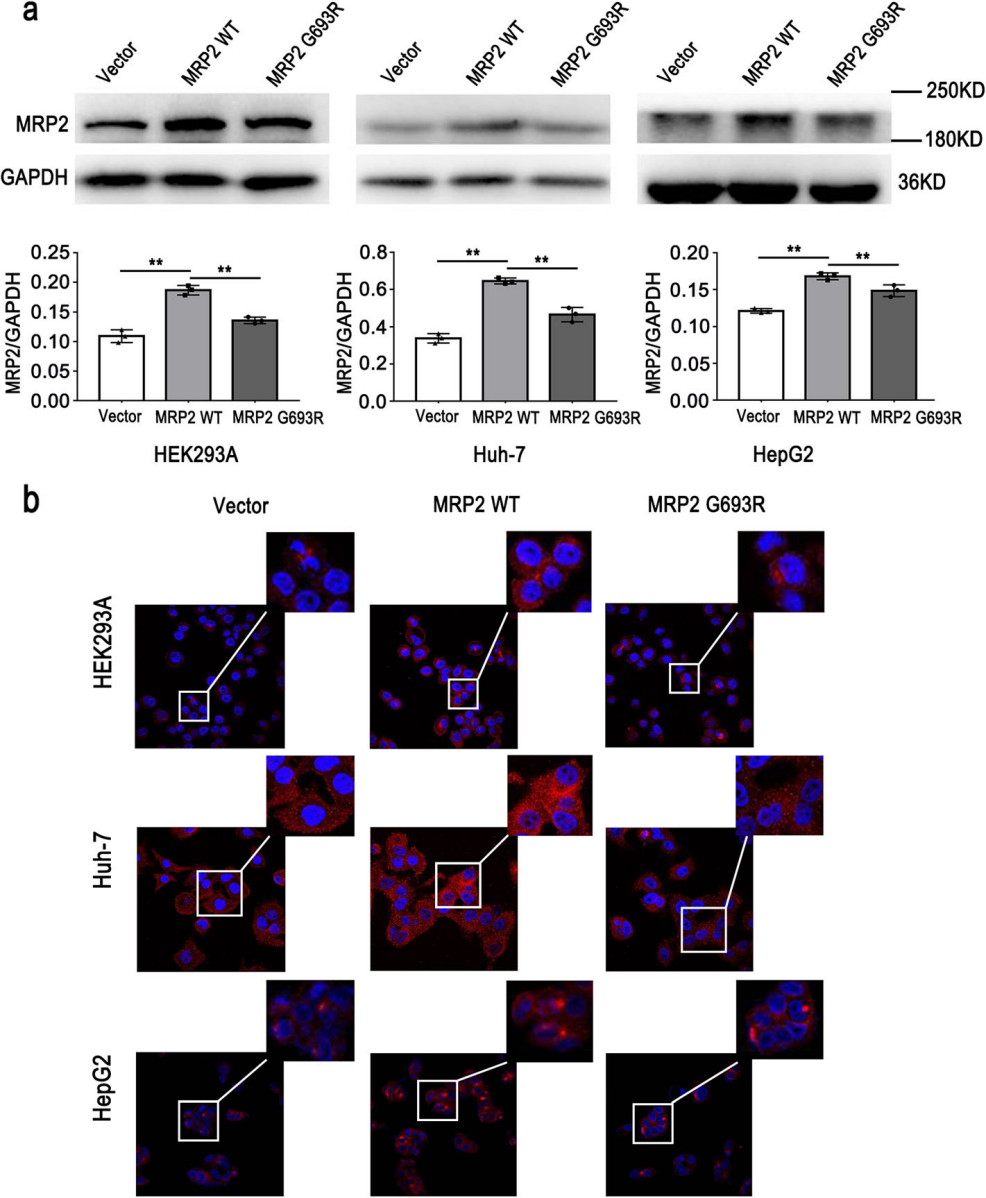
The MRP2 p.G693R mutant showed decreased expression and mislocalization in three cell lines in vitro. **a** Western blotting demonstrated significantly decreased expression of MRP2 in three cell lines expressing the p.G693R mutant compared with those expressing wild-type MRP2. ^**^P < 0.05. **b** Immunofluorescence assay showed the mislocalization of the MRP2 p.G693R mutant, which was predominantly retained in the cytoplasm rather than the cell surface

Western blotting for MRP2 in HEK293, Huh-7 and HepG2 cell lines transfected with vector expressing *ABCC2* with the p.G693R mutation demonstrated a decreased expression of the MRP2 p.G693R mutant, probably due to the degradation of the mutant protein, compared with wild-type MRP2 (*p* < 0.01) (Fig. 2a).

### MRP2 p.G693R mutant was predominantly retained in the cytoplasm rather than the cell surface

**Fig. 3:**
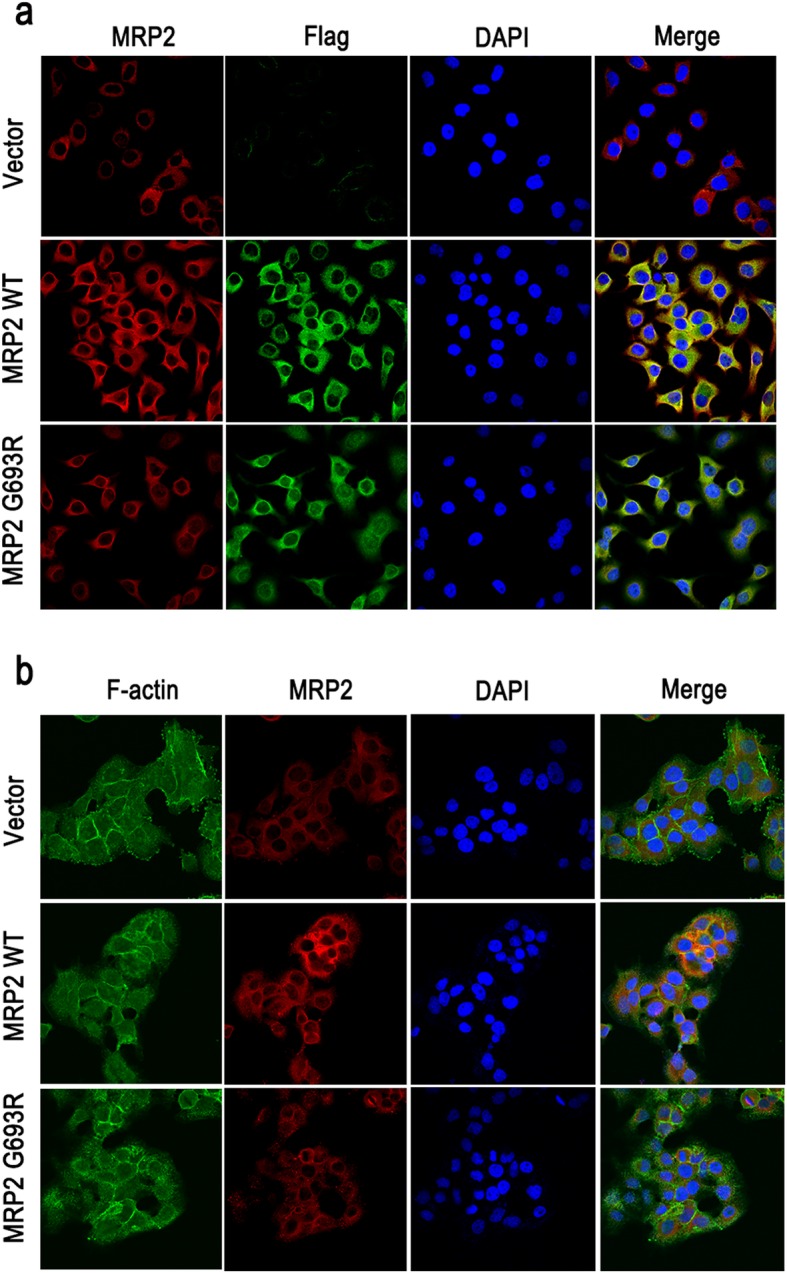
The MRP2 p.G693R mutant with flag tag showed mislocalization in Huh-7 cells in vitro. **a** Immunofluorescence analysis of MRP2 fusion protein with N-terminal flag tag in Huh-7 cells confirmed MRP2 expression in wild-type and p.G693R mutant MRP2 plasmids. **b** Immunofluorescence analysis of F-actin and MRP2 expression in Huh-7 cells showed wild-type MRP2 predominantly localized to the cell surface and cytoplasm, while MRP2 p.G693R mutant was mostly retained at the cytoplasm

Indirect immunofluorescence staining in HEK293A, Huh-7 and HepG2 cell lines showed that wild-type MRP2 predominantly localized to the cell surface and cytoplasm. In contrast, we observed mislocalization of the MRP2 p.G693R mutant, which was predominantly retained in the cytoplasm rather than the cell surface (Fig. 2b). Immunofluorescence analysis of MRP2 fusion protein with N-terminal flag tag, and F-actin in Huh-7 cells showed wild-type MRP2 predominantly localized to the cell surface and cytoplasm, while MRP2 p.G693R mutant was mostly retained at the cytoplasm (Fig. 3a-b).

### MRP2 p.G693R mutant exhibited decreased organic anion transport activity

**Fig. 4:**
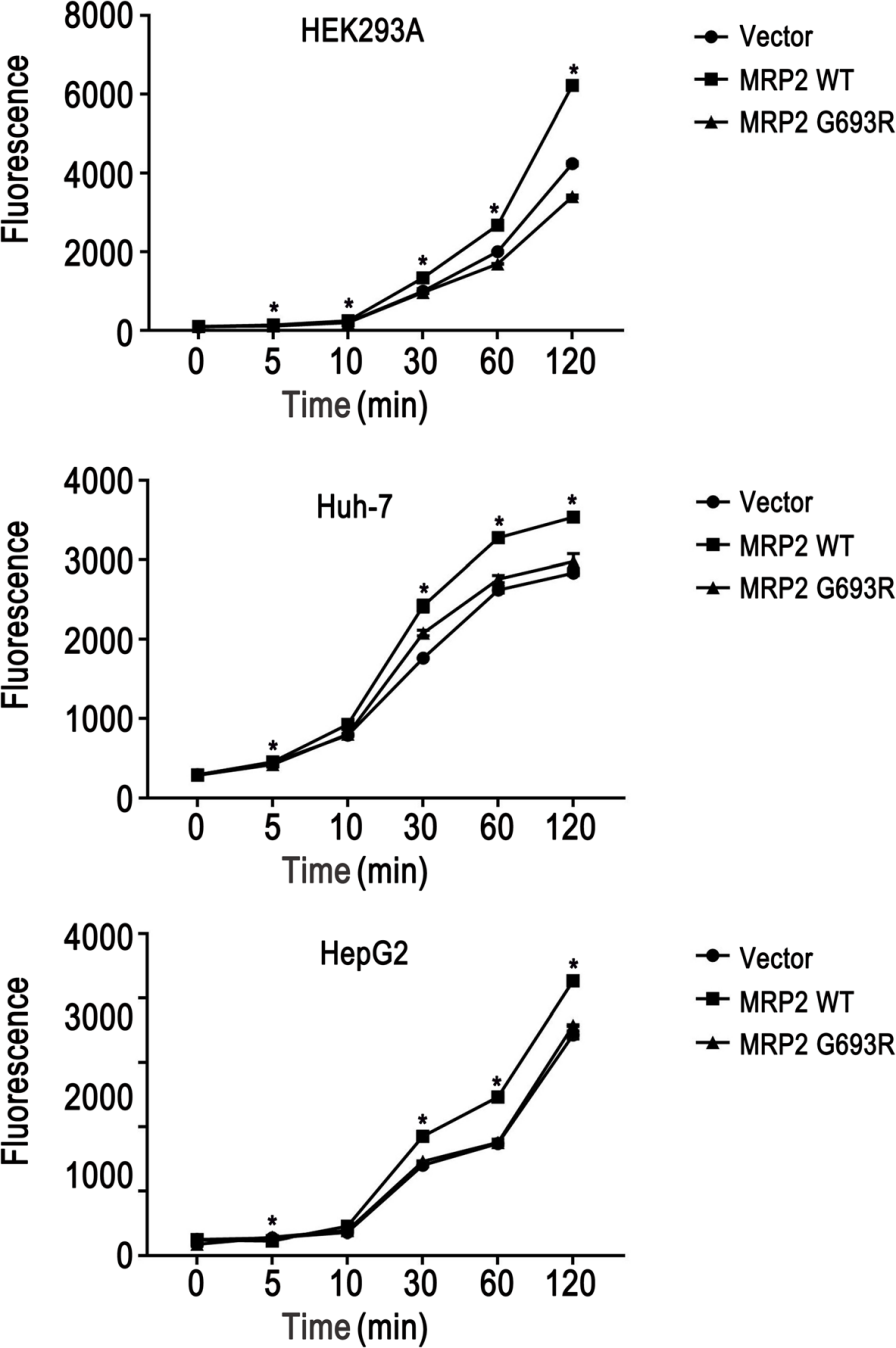
MRP2 p.G693R mutant exhibited decreased organic anion transport activity. Compared with wild-type MRP2, the MRP2 p.G693R mutant exhibited decreased organic anion transport activity in HEK293A, Huh-7 and HepG2 cell lines. The Student’s test was used to evaluate the differences of organic anion transport activity. ^*^P < 0.05

Following pre-incubation with MCLB, which was conjugated with glutathione to produce GS-MCLB, the GS-MCLB efflux in HEK293A, Huh-7 and HepG2 cells lines which expressed wild-type MRP2 was markedly increased compared with that in cells expressed MRP2 p.G693R (*p* < 0.05) (Fig. 4). These results indicated that the p.G693R mutant exhibited significantly decreased organic anion transport activity compared with the wild-type MRP2.

## Discussion

In the present study, we reported on the clinical manifestations and biological functional consequences of the *ABCC2* p.G693R mutation, which was identified with a high frequency of 28.6% (2/7) in a small cohort of Chinese patients with DJS. Functional studies indicated significantly decreased expression, mislocalization, and decreased organic anion transport activity of mutant MRP2 compared with wild-type MRP2, indicating that this variant resulted in loss of function of the MRP2 protein.

DJS is characterized by predominantly conjugated hyperbilirubinemia in liver function tests and black liver in liver biopsies [11]. Urinary coproporphyrin levels, bromsulphalein excretion test and hepatobiliary scintigraphy with iodopanoic acid provide effective methods for identification and diagnosis on DJS [12, 13]. However, most of the diagnostic tests, including urinary corproporphyrin or bromsulphalein excretion test, are not widely available in clinical practice in China. Diagnose of DJS relies of *ABCC2* gene Sanger sequencing or liver histopathology manifestations. Since liver biopsies are invasive, genetic analysis of peripheral blood samples is important. To date, a total of 68 *ABCC2* variants have been reported in DJS according to HGMD, comprising 31 (45.6%) missense variants, 13 (19.1%) small deletions variants, 10 (14.7%) nonsense variants, 6 (8.8%) splice site variants and 3 (4.4%) gross deletions variants, 2 (2.9%) small indels variants, 2 (2.9%) insertions variants and 1 (1.5%) gross insertion variant, and the variants occur randomly throughout the gene (Supplementary Table 1). Of the 31 missense variants, 14 are pathogenic confirmed by previous functional study [14–21]. As the clinical data including serum level of total bilirubin were not available for the individual missense mutation carriers in the previous studies, the genotype and phenotype correlation remains to be explored. However, in the present study, the abnormal serum level of total bilirubin of 94 and 97 μmol/L were observed in the two cases with *ABCC2* G693R mutation respectively. Therefore, identification and functional analysis of causative *ABCC2* gene mutations for patients suspected with DJS are essential in diagnosis of DJS in China.

In the present study, whole exome sequencing revealed additional *HSD3B7* p.R110Q mutation in a case with *ABCC2* p.G693R mutation, but no mutations in the other known hyperbilirubinemia-related genes in the other case with *ABCC2* p.G693R mutation. Gene *HSD3B7*, located on chromosome 16p11.2, encodes an enzyme which is a member of the short-chain dehydrogenase/reductase superfamily and involved in the initial stages of the synthesis of bile acids from cholesterol [22]. Pathogenic mutations in *HSD3B7* are associated with congenital bile acid deficiency type I, which is a life-threatening liver disease and manifests as hyperbilirubinemia and neonatal cholestasis [23, 24]. However, in silico analysis showed this variant was benign, indicating it might be common variant but not a pathogenic mutation. Thus, the two cases with *ABCC2* p.G693R heterozygous mutation showed that the *ABCC2* mutation might be the main genetic factor of DJS in China.

In the present study, the p.G693R mutation was observed in 2 out of 7 patients, indicating that the p.G693R mutation might be a potential hotspot mutation in Chinese DJS patients. *ABCC2* p.G693R is a missense mutation located in the first ATP-binding domain of MRP2 protein [25]. We demonstrated that while wild-type MRP2 predominantly localized to the cell surface and cytoplasm, the MRP2 p.G693R mutant was predominantly retained in the cytoplasm rather than the cell surface. The mislocalization of the p.G693R mutant may likely be due to deficient maturation and sorting, causing impaired insertion trafficking from the endoplasmic reticulum to the canalicular membrane in hepatocytes, similar to the p.R768W and p.W709R mutants reported in previous studies [26, 27].

Furthermore, efflux of GS-MCLB uptake into plasma from cells expressing the p.G693R mutant was markedly reduced compared with cells expressing wild-type MRP2, suggesting that the organic anion transport activity of p.G693R MRP2 on the canalicular membrane was defective [13]. Similarly, Hashimoto et al. reported that p.Q1382R MRP2 impairs substrate-induced ATP hydrolysis, which lead to the defect of organic anion transport activity [6].

We understand that the number of cases analyzed in the current study was limited, and thus more cases with DJS are required to confirm these conclusions. Using the China Registry of Genetic/Metabolic Liver Diseases, we will conduct further genetic and functional studies of DJS to strengthen the relationship between the genotype and phenotype of DJS.

## Conclusions

In conclusion, here we show that the *ABCC2* p.G693R mutation causes dysfunction of the MRP2 protein and may result in hyperbilirubinemia in DJS in China.

## Supplementary information

**Additional file 1: Supplementary Table S1.** Known variants identified in the *ABCC2* gene according to Human Gene Mutation Database.

## Data Availability

All the data were collected from the hospital information system and can be available from the corresponding author upon reasonable request.

## Abbreviations

*ABCC2*: Adenosine triphosphate-binding cassette subfamily C member 2
DJS: Dubin-Johnson syndrome
GS-MCLB: Glutathione conjugated monochlorobimane
HEK: Human embryonic kidney
HGMD: Human Gene Mutation Database
HRP: Horseradish peroxidase
*HSD3B7*: 3β-hydroxysteroid dehydrogenase type 7
MRP2: Multidrug resistance-associated protein 2
SD: Standard deviation
