# Supplementary material for: A recurrent ABCC2 p.G693R mutation resulting in loss of function of MRP2 and hyperbilirubinemia in Dubin-Johnson syndrome in China
Source: Orphanet J Rare Dis. 2020 Mar 18;15:74. doi: 10.1186/s13023-020-1346-4 (PMC7079413; doi:10.1186/s13023-020-1346-4)
Supplement: Supplementary file 1 — Additional file 1: Supplementary Table S1. Known variants identified in the ABCC2 gene according to Human Gene Mutation Database. [file 13023_2020_1346_MOESM1_ESM.docx]

| **Supplementary Table** **1** Known variants identified in the *ABCC2* gene according to Human Gene Mutation Database | | | | | | | | | | | | | |
| --- | --- | --- | --- | --- | --- | --- | --- | --- | --- | --- | --- | --- | --- |
| Gene | Location | Base change | Amino acid change | Variation Types | Allele Frequency (Total) | Allele Frequency (East Asian) | SIFT Prediction | Polyphen-2 Prediction | MutationTaster Prediction | TB level (mg/dl) | Biological functional consequence | References |  |
| *ABCC2* | Exon 3 | c.298C>T | p.R100X | Nonsense | 3.656e-05 | 1e-04 | NA | NA | Disease causing | 3.2 | NA | (1) |  |
| *ABCC2* | Exon 4 | c.334-2_337delAGCTCC | p.L112Wfs*4/Splicing | Small deletion | NA | NA | NA | NA | NA | 1.6 | NA | (2) |  |
| *ABCC2* | Exon 4 | c.351_355dupCCAAT | p.Q118Pfs*35 | Small insertion | NA | NA | NA | NA | NA | 16.4 | NA | (3) |  |
| *ABCC2* | Exon 5 | c.537_542delGATCTT | p.I180-; p.F181- | Small deletion | NA | NA | NA | NA | NA | 2 | NA | (2) |  |
| *ABCC2* | Exon 7 | c.821_822delCT | p.P274Rfs*19 | Small deletion | NA | NA | NA | NA | NA | 6.4 | NA | (4) |  |
| *ABCC2* | Exon 8 | c.974C>G | p.S325X | Nonsense | 2.844e-05 | 0 | NA | NA | Disease causing | 3 | NA | (5) |  |
| *ABCC2* | Exon 8 | c.998A>G | p.D333G | Missense | 3e-04 | 0 | Damaging | Probably damaging | Disease causing | NA | Decreased expression | (6) |  |
| *ABCC2* | Exon 8 | c.1013_1014delTG | p.V338Efs*14 | Small deletion | NA | NA | NA | NA | NA | 4.1 | NA | (7) |  |
| *ABCC2* | Exon 9 | c.1135C>A | p.Q379K | Missense | NA | NA | Tolerable | Probably damaging | Disease causing | 6.4 | NA | (4) |  |
| *ABCC2* | Exon 9 | c.1177C>T | p.R393W | Missense | 7.321e-05 | 6e-04 | Damaging | Probably damaging | Disease causing | 2.3 | NA | (8) |  |
| *ABCC2* | Exon 10 | c.1234A>G | p.R412G | Missense | 4.065e-06 | 0 | Damaging | Probably damaging | Disease causing | NA | Decreased transport activity | (9) |  |
| *ABCC2* | Exon 10 | c.1249G>A | p.V417I | Missense | 1.917e-01 | 8.75e-02 | Tolerable | Benign | Polymorphism | 4.5 | Decreased transport activity | (10) |  |
| *ABCC2* | Exon 10 | c.1256_1272delAAACAGTGAACCTGATGinsCT | p.E419A; p.T420-; p.V421-; p.N422-; p.L423-; p.M424- | Small indel | NA | NA | NA | NA | NA | 5.6 | NA | (11) |  |
| *ABCC2* | Exon 10 | c.1303A>C | p.T435P | Missense | NA | NA | Damaging | Probably damaging | Disease causing | 1.8 | NA | (12) |  |
| *ABCC2* | Exon 10 | c.1321C>A | p.L441M | Missense | 4.065e-06 | 5.801e-05 | Damaging | Probably damaging | Disease causing | 8 | NA | (13) |  |
| *ABCC2* | Exon 10 | c.1326G>A | p.W442X | Nonsense | NA | NA | NA | NA | Disease causing | 1.8 | NA | (12) |  |
| *ABCC2* | Exon 10 | c.1342A>G | p.I448V | Missense | NA | NA | Tolerable | Probably damaging | Disease causing | 6.1 | NA | (14) |  |
| *ABCC2* | Exon 10 | c.1354_1357delATCT | p.I452Sfs*12 | Small deletion | NA | NA | NA | NA | NA | 11 | NA | (3) |  |
| *ABCC2* | Exon 15 | c.1939G>T | p.E647X | Nonsense | 4.067e-06 | 0 | NA | NA | Disease causing | 5.3 | NA | (15) |  |
| *ABCC2* | Exon 16 | c.2026G>C | p.G676R | Missense | NA | NA | Damaging | Probably damaging | Disease causing | 3.4 | NA | (16) |  |
| *ABCC2* | Exon 16 | c.2077G>A | p.G693R | Missense | 3.662e-05 | 0 | Damaging | Probably damaging | Disease causing | 5.7 | NA | (15) |  |
| *ABCC2* | Exon 17 | c.2125T>C | p.W709R | Missense | 4.064e-06 | 5.801e-05 | Damaging | Probably damaging | Disease causing | 2.6 | NA | (17) |  |
| *ABCC2* | Exon 18 | c.2273G>T | p.G758V | Missense | 4.064e-06 | 0 | Damaging | Probably damaging | Disease causing | NA | NA | (18) |  |
| *ABCC2* | Exon 18 | c.2302C>T | p.R768W | Missense | 9.345e-05 | 6e-04 | Damaging | Probably damaging | Disease causing | 5 | NA | (19) |  |
| *ABCC2* | Exon 18 | c.2325delC | p.Y776Tfs*4 | Small deletion | NA | NA | NA | NA | NA | 6.74 | NA | (20) |  |
| *ABCC2* | Exon 18 | c.2360_2366delCCCTGTC | p.P787Lfs*7 | Small deletion | NA | NA | NA | NA | NA | 12.9 | NA | (21) |  |
| *ABCC2* | Exon 18 | c.2366C>T | p.S789F | Missense | 6e-04 | 8.2e-03 | Damaging | Probably damaging | Disease causing | NA | Decreased expression, mislocalization, decreased transport activity | (22) |  |
| *ABCC2* | Exon 18 | c.2423G>T | p.G808V | Missense | NA | NA | Damaging | Probably damaging | Disease causing | 5.7 | NA | (15) |  |
| *ABCC2* | Exon 18 | c.2426delT | p.L809Rfs*2 | Small deletion | NA | NA | NA | NA | NA | NA | NA | (23) |  |
| *ABCC2* | Exon 19 | c.2561_2562delAG | p.E854Vfs*3 | Small deletion | NA | NA | NA | NA | NA | NA | NA | (24) |  |
| *ABCC2* | Exon 20 | c.2736_2737delAC | p.L913X | Small deletion | NA | NA | NA | NA | NA | 1.6 | NA | (2) |  |
| *ABCC2* | Exon 21 | c.2882A>G | p.K961R | Missense | 1.636e-05 | 2e-04 | Tolerable | Benign | Disease causing | NA | NA | (25) |  |
| *ABCC2* | Exon 22 | c.2901C>A | p.Y967X | Nonsense | 2.032e-05 | 0 | NA | NA | Disease causing | NA | NA | (26) |  |
| *ABCC2* | Exon 23 | c.3196C>T | p.R1066X | Nonsense | 4e-04 | 0 | NA | NA | Disease causing | 4.1 | NA | (27) |  |
| *ABCC2* | Exon 23 | c.3258+2dupT | Indel/splice | Small insertion | NA | NA | NA | NA | NA | NA | NA | (28) |  |
| *ABCC2* | Exon 24 | c.3305G>A | p.W1102X | Nonsense | 4.061e-06 | 0 | NA | NA | Disease causing | NA | NA | (26) |  |
| *ABCC2* | Exon 24 | c.3399_3400delTT | p.Y1134Cfs*43 | Small deletion | NA | NA | NA | NA | NA | 8.1 | NA | (13) |  |
| *ABCC2* | Exon 25 | c.3449G>A | p.R1150H | Missense | 9.746e-05 | 6.534e-05 | Damaging | Probably damaging | Disease causing | NA | Decreased transport activity | (29) |  |
| *ABCC2* | Exon 25 | c.3517A>T | p.I1173F | Missense | 3.655e-05 | 0 | Damaging | Probably damaging | Disease causing | NA | Decreased expression, mislocalization, decreased transport activity | (29) |  |
| *ABCC2* | Exon 25 | c.3521G>A | p.R1174H | Missense | 2e-04 | 1e-04 | Damaging | Probably damaging | Disease causing | NA | Decreased expression, mislocalization, decreased transport activity | (6) |  |
| *ABCC2* | Exon 25 | c.3542G>T | p.R1181L | Missense | 7.4e-03 | 0 | Damaging | Probably damaging | Disease causing | NA | Decreased expression, decreased transport activity | (6) |  |
| *ABCC2* | Exon 25 | c.3563T>A | p.V1188E | Missense | 4.5e-02 | 8e-04 | Tolerable | Benign | Polymorphism | NA | Decreased transport activity | (6, 30) |  |
| *ABCC2* | Exon 26 | c.3732T>G | p.N1244K | Missense | 3.655e-05 | 4e-04 | Damaging | Probably damaging | Disease causing | NA | Decreased transport activity | (6) |  |
| *ABCC2* | Exon 27 | c.3817A>G | p.T1273A | Missense | 9e-04 | 0 | Damaging | Benign | Polymorphism | 3 | NA | (5) |  |
| *ABCC2* | Exon 27 | c.3825C>G | p.Y1275X | Nonsense | 6.497e-05 | 9e-04 | NA | NA | Disease causing | 9.6 | NA | (13) |  |
| *ABCC2* | Exon 28 | c.3872C>T | p.P1291L | Missense | 2.8e-03 | 0 | Damaging | Probably damaging | Disease causing | NA | Decreased transport activity | (6) |  |
| *ABCC2* | Exon 28 | c.3928C>T | p.R1310X | Nonsense | 2.031e-05 | 2e-04 | NA | NA | Disease causing | 3 | NA | (31) |  |
| *ABCC2* | Exon 29 | c.4025C>A | p.S1342Y | Missense | NA | NA | Damaging | Probably damaging | Disease causing | NA | NA | (32) |  |
| *ABCC2* | Exon 29 | c.4055A>C | p.E1352A | Missense | NA | NA | Damaging | Probably damaging | Disease causing | 0.6 | NA | (33) |  |
| *ABCC2* | Exon 29 | c.4054G>C | p.E1352Q | Missense | NA | NA | Damaging | Probably damaging | Disease causing | 16.3 | NA | (13) |  |
| *ABCC2* | Exon 29 | c.4145A>G | p.Q1382R | Missense | NA | NA | Damaging | Probably damaging | Disease causing | 2.5 | NA | (34) |  |
| *ABCC2* | Exon 30 | c.4175_4180delGGATGA | p.R1392-: p.M1393- | Small deletion | NA | NA | NA | NA | NA | 2.75 | NA | (35) |  |
| *ABCC2* | Exon 30 | c.4292_4293delCA | p.T1431Rfs*31 | Small deletion | NA | NA | NA | NA | NA | 5.6 | NA | (11) |  |
| *ABCC2* | Exon 31 | c.4327C>T | p.Q1443X | Nonsense | 8.132e-06 | 0 | NA | NA | Disease causing | NA | NA | (26) |  |
| *ABCC2* | Exon 31 | c.4348G>A | p.A1450T | Missense | NA | NA | Damaging | Probably damaging | Disease causing | NA | Decreased expression, mislocalization, decreased transport activity | (22) |  |
| *ABCC2* | Exon 31 | c.4430C>T | p.T1477M | Missense | 1.2e-03 | 1e-04 | Tolerable | Benign | Polymorphism | NA | Decreased transport activity | (36) |  |
| *ABCC2* | Exon 31 | c.4465_4473delATCACCATCinsGGCCCACAG | p.I1489G; p.T1490P; p.I1491Q | Small indel | NA | NA | NA | NA | NA | 6.1 | NA | (14) |  |
| *ABCC2* | Exon 32 | c.4544G>A | p.C1515Y | Missense | 5.21e-02 | 8e-04 | Tolerable | Benign | Polymorphism | NA | Decreased transport activity | (6, 37) |  |
| *ABCC2* | 5'-UTR | c.-24C>T |  | Splicing | NA | NA | NA | NA | NA | NA | NA | (38) |  |
| *ABCC2* | Intron 8-9 | c.1031+4A>G |  | Splicing | NA | NA | NA | NA | NA | NA | NA | (39) |  |
| *ABCC2* | Intron 13-14 | c.1815+2T>A |  | Splicing | NA | NA | NA | NA | NA | 5.2 | NA | (19) |  |
| *ABCC2* | Intron 15-16 | c.1967+2T>C |  | Splicing | NA | NA | NA | NA | NA | 4 | NA | (40) |  |
| *ABCC2* | Intron 18-19 | c.2439+2T>C |  | Splicing | NA | NA | NA | NA | NA | 1.3 | NA | (19) |  |
| *ABCC2* | Intron 23-24 | c.3258+1G>A |  | Splicing | NA | NA | NA | NA | NA | 12.9 | NA | (21) |  |
| *ABCC2* |  | deletion 136 bp c.2748 |  | Gross deletion | NA | NA | NA | NA | NA | 9.6 | NA | (13) |  |
| *ABCC2* |  | deletion 229 bp c.3615 |  | Gross deletion | NA | NA | NA | NA | NA | 16.4 | NA | (13) |  |
| *ABCC2* |  | deletion 1008 bp incl. ex. 7 |  | Gross deletion | NA | NA | NA | NA | NA | 4 | NA | (41) |  |
| *ABCC2* |  | insertion 5299 bp incl. ex. 24-25 |  | Gross insertion | NA | NA | NA | NA | NA | 0.6 | NA | (33) |  |
| TB: Total bilirubin; SIFT: sorting intolerant from tolerant; NA: not available. | | | | | | | | | | | | | |

**References**

1. Shoda J. Novel mutations identified in the human multidrug resistance-associated protein 2 (MRP2/ABCC2) gene in a Japanese patient with Dubin–Johnson syndrome. Hepatology Research 2003;27:323-326.

2. Huynh MT, Chrétien Y, Grison S, Delaunay JL, Lascols O, Tran CT, Goria O, et al. Novel compound heterozygous ABCC2 variants in patients with Dubin-Johnson syndrome and intrahepatic cholestasis of pregnancy. Clinical Genetics 2018;94:480-481.

3. Togawa T, Mizuochi T, Sugiura T, Kusano H, Tanikawa K, Sasaki T, Ichinose F, et al. Clinical, Pathologic, and Genetic Features of Neonatal Dubin-Johnson Syndrome: A Multicenter Study in Japan. J Pediatr 2018, 28. doi: 10.1016/j.jpeds.2017.12.058.

4. Devgun MS, El-Nujumi AM, O'Dowd GJ, Barbu V, Poupon R. Novel mutations in the Dubin-Johnson syndrome gene ABCC2/MRP2 and associated biochemical changes. Ann Clin Biochem 2012;49:609-612.

5. Corpechot C, Ping C, Wendum D, Matsuda F, Barbu V, Poupon R. Identification of a novel 974C-->G nonsense mutation of the MRP2/ABCC2 gene in a patient with Dubin-Johnson syndrome and analysis of the effects of rifampicin and ursodeoxycholic acid on serum bilirubin and bile acids. Am J Gastroenterol 2006;101:2427-2432.

6. Arlanov R, Porter A, Strand D, Brough R, Karpova D, Kerb R, Wojnowski L, et al. Functional characterization of protein variants of the human multidrug transporter ABCC2 by a novel targeted expression system in fibrosarcoma cells. Hum Mutat 2012;33:750-762.

7. Slachtova L, Seda O, Behunova J, Mistrik M, Martasek P. Genetic and biochemical study of dual hereditary jaundice: Dubin-Johnson and Gilbert's syndromes. Haplotyping and founder effect of deletion in ABCC2. Eur J Hum Genet 2016;24:704-709.

8. Machida I, Wakusawa S, Sanae F, Hayashi H, Kusakabe A, Ninomiya H, Yano M, et al. Mutational analysis of the MRP2 gene and long-term follow-up of Dubin-Johnson syndrome in Japan. J Gastroenterol 2005;40:366-370.

9. Hulot JS, Villard E, Maguy A, Morel V, Mir L, Tostivint I, William-Faltaos D, et al. A mutation in the drug transporter gene ABCC2 associated with impaired methotrexate elimination. Pharmacogenet Genomics 2005; 15:277-285.

10. Meyer zu Schwabedissen HE, Jedlitschky G, Gratz M, Haenisch S, Linnemann K, Fusch C, Cascorbi I, et al. Variable expression of MRP2 (ABCC2) in human placenta: influence of gestational age and cellular differentiation. Drug Metab Dispos 2005;33:896-904.

11. Cebecauerova D, Jirasek T, Budisova L, Mandys V, Volf V, Novotna Z, Subhanova I, et al. Dual hereditary jaundice: simultaneous occurrence of mutations causing Gilbert's and Dubin-Johnson syndrome. Gastroenterology 2005;129:315-320.

12. Xiang R, Li JJ, Fan LL, Jin JY, Xia K, Wang F. Identification of a compound heterozygous mutation of ABCC2 in a patient with hyperbilirubinemia. Mol Med Rep 2017;16:2830-2834.

13. Lee JH, Chen HL, Chen HL, Ni YH, Hsu HY, Chang MH. Neonatal Dubin-Johnson syndrome: long-term follow-up and MRP2 mutations study. Pediatr Res 2006;59:584-589.

14. Jiang J, Wang HG, Wu WL, Peng XX. Mixed Dubin-Gilbert Syndrome: A Compound Heterozygous Phenotype of Two Novel Variants in ABCC2 Gene. Chin Med J (Engl) 2017;130:1003-1005.

15. Wu L, Zhang W, Jia S, Zhao X, Zhou D, Xu A, Duan W, et al. Mutation analysis of the gene in Chinese patients with Dubin-Johnson syndrome. Exp Ther Med 2018;16:4201-4206.

16. Wakusawa S, Machida I, Suzuki S, Hayashi H, Yano M, Yoshioka K. Identification of a novel 2026G-->C mutation of the MRP2 gene in a Japanese patient with Dubin-Johnson syndrome. J Hum Genet 2003;48:425-429.

17. Machida I, Inagaki Y, Suzuki S, Hayashi H, Wakusawa S. Mutation analysis of the multidrug resistance protein 2 (MRP2) gene in a Japanese patient with Dubin-Johnson syndrome. Hepatol Res 2004;30:86-90.

18. Abouelhoda M, Sobahy T, El-Kalioby M, Patel N, Shamseldin H, Monies D, Al-Tassan N, et al. Clinical genomics can facilitate countrywide estimation of autosomal recessive disease burden. Genet Med 2016;18:1244-1249.

19. Wada M, Toh S, Taniguchi K, Nakamura T, Uchiumi T, Kohno K, Yoshida I, et al. Mutations in the canalicular multispecific organic anion transporter (cMOAT) gene, a novel ABC transporter, in patients with hyperbilirubinemia II/Dubin–Johnson syndrome. Hum Mol Genet 1998; 7: 203–207.

20. Erlinger S, Arias IM, Dhumeaux D. Inherited disorders of bilirubin transport and conjugation: new insights into molecular mechanisms and consequences. Gastroenterology 2014;146:1625-1638.

21. Sticova E, Elleder M, Hulkova H, Luksan O, Sauer M, Wunschova-Moudra I, Novotny J, et al. Dubin-Johnson syndrome coinciding with colon cancer and atherosclerosis. World J Gastroenterol 2013;19:946-950.

22. Hirouchi M, Suzuki H, Itoda M, Ozawa S, Sawada J, Ieiri I, Ohtsubo K, Sugiyama Y. Characterization of the Cellular Localization, Expression Level, and Function of SNP Variants of MRP2/ABCC2. Pharm Res 2004; 21:742-748.

23. Abuli A, Boada M, Rodriguez-Santiago B, Coroleu B, Veiga A, Armengol L, Barri PN, et al. NGS-Based Assay for the Identification of Individuals Carrying Recessive Genetic Mutations in Reproductive Medicine. Hum Mutat 2016;37:516-523.

24. Alfares AA. Applying filtration steps to interpret the results of whole-exome sequencing in a consanguineous population to achieve a high detection rate. Int J Health Sci (Qassim) 2018;12:35-43.

25. Togawa T, Sugiura T, Ito K, Endo T, Aoyama K, Ohashi K, Negishi Y, et al. Molecular Genetic Dissection and Neonatal/Infantile Intrahepatic Cholestasis Using Targeted Next-Generation Sequencing. J Pediatr 2016;171:171-177.

26. Tabor HK, Auer PL, Jamal SM, Chong JX, Yu JH, Gordon AS, Graubert TA, et al. Pathogenic variants for Mendelian and complex traits in exomes of 6,517 European and African Americans: implications for the return of incidental results. Am J Hum Genet 2014;95:183-193.

27. Paulusma CC, Kool M, Bosma PJ, Scheffer GL, ter Borg F, Scheper RJ, Tytgat GN, et al. A mutation in the human canalicular multispecific organic anion transporter gene causes the Dubin-Johnson syndrome. Hepatology 1997; 25:1539-1542.

28. Motazacker MM, Peter J, Treskes M, Shoulders CC, Kuivenhoven JA, Hovingh GK. Evidence of a polygenic origin of extreme high-density lipoprotein cholesterol levels. Arterioscler Thromb Vasc Biol 2013;33:1521-1528.

29. Mor-Cohen R, Zivelin A, Rosenberg N, Shani M, Muallem S, Seligsohn U. Identification and functional analysis of two novel mutations in the multidrug resistance protein 2 gene in Israeli patients with Dubin-Johnson syndrome. J Biol Chem 2001;276:36923-36930.

30. Floreani A, Carderi I, Variola A, Rizzotto ER, Nicol J, Bergasa NV. A novel multidrug-resistance protein 2 gene mutation identifies a subgroup of patients with primary biliary cirrhosis and pruritus. Hepatology 2006;43:1152-1154.

31. Tate G, Li M, Suzuki T, Mitsuya T. A new mutation of the ATP-binding cassette, sub-family C, member 2 (ABCC2) gene in a Japanese patient with Dubin-Johnson syndrome. Genes Genet Syst 2002; 77: 117-121.

32. Kruer MC, Jepperson T, Dutta S, Steiner RD, Cottenie E, Sanford L, Merkens M, et al. Mutations in gamma adducin are associated with inherited cerebral palsy. Ann Neurol 2013;74:805-814.

33. Dixon PH, Sambrotta M, Chambers J, Taylor-Harris P, Syngelaki A, Nicolaides K, Knisely AS, et al. An expanded role for heterozygous mutations of ABCB4, ABCB11, ATP8B1, ABCC2 and TJP2 in intrahepatic cholestasis of pregnancy. Sci Rep 2017;7:11823.

34. Toh S, Wada M, Uchiumi T, Inokuchi A, Makino Y, Horie Y, Adachi Y, et al. Genomic structure of the canalicular multispecific organic anion-transporter gene (MRP2/cMOAT) and mutations in the ATP-binding-cassette region in Dubin-Johnson syndrome. Am J Hum Genet 1999;64:739-746.

35. Tsujii H, König J, Rost D, Stöckel B, Leuschner U, Keppler D. Exon-intron organization of the human multidrug-resistance protein 2 (MRP2) gene mutated in Dubin-Johnson syndrome. Gastroenterology 1999;117:653-660.

36. Megaraj V, Zhao T, Paumi CM, Gerk PM, Kim RB, Vore M. Functional analysis of nonsynonymous single nucleotide polymorphisms of multidrug resistance-associated protein 2 (ABCC2). Pharmacogenet Genomics 2011;21:506-515.

37. Elens L, Tyteca D, Panin N, Courtoy P, Lison D, Demoulin JB, Haufroid V. Functional defect caused by the 4544G>A SNP in ABCC2: potential impact for drug cellular disposition. Pharmacogenet Genomics 2011;21:884-893.

38. Ufer M, Mosyagin I, Muhle H, Jacobsen T, Haenisch S, Hasler R, Faltraco F, et al. Non-response to antiepileptic pharmacotherapy is associated with the ABCC2 -24C>T polymorphism in young and adult patients with epilepsy. Pharmacogenet Genomics 2009;19:353-362.

39. Mor-Cohen R, Zivelin A, Rosenberg N, Goldberg I, Seligsohn U. A novel ancestral splicing mutation in the multidrug resistance protein 2 gene causes Dubin-Johnson syndrome in Ashkenazi Jewish patients. Hepatol Res 2005;31:104-111.

40. Kajihara S, Hisatomi A, Mizuta T, Hara T, Ozaki I, Wada I, Yamamoto K. A splice mutation in the human canalicular multispecific organic anion transporter gene causes Dubin-Johnson syndrome. Biochem Biophys Res Commun 1998;253:454-457.

41. Kanda D, Takagi H, Kawahara Y, Yata Y, Takakusagi T, Hatanaka T, Yoshinaga T, et al. Novel large-scale deletion (whole exon 7) in the ABCC2 gene in a patient with the Dubin-Johnson syndrome. Drug Metab Pharmacokinet 2009;24:464-468.
